# Time for action: Intimate partner violence troubles one third of Ethiopian women

**DOI:** 10.1371/journal.pone.0216962

**Published:** 2019-05-16

**Authors:** Kiddus Yitbarek, Mirkuzie Woldie, Gelila Abraham

**Affiliations:** 1 Department of Health Policy and Management, Institute of Health, Jimma University, Jimma, Ethiopia; 2 Department of Global Health and Population, Harvard T.H. Chan School of Public Health, Boston, Massachusetts, United States of America; Anglia Ruskin University, UNITED KINGDOM

## Abstract

**Background:**

Intimate partner violence is a major challenges faced by women especially in developing world. Its consequences range from personal health problems up to countrywide loss of productivity and poverty. There is limited empirical evidence documenting intimate partner violence and underlying reasons in Ethiopia. Therefore, the aim of this study was to assess the magnitude of intimate partner violence and associated factors in Ethiopia.

**Methods:**

We analyzed the 2016 Ethiopian demographic and health survey data. About 2,750 women aged 15–49 years were included in the survey. Intimate partner violence was measured in three dimensions: physical, emotional and sexual violence. Multiple logistic regression was conducted to identify independent predictors. Variables with p-value less than 0.05 were considered as significantly associated with dimensions of violence. All analysis were adjusted for clusters and sample weights.

**Results:**

Overall 32.5% of Ethiopian women experienced at least one type of intimate partner violence. Physical and emotional violence were each experienced by 22.5% of the women, while 9.6% of the study participants encountered sexual violence. The age difference between a woman and her intimate partner has a positive effect on emotional and sexual violence while the opposite is true for physical violence. Moreover, physical violence was significantly associated with place of residence, and husband education. Both emotional and sexual violence were predicted by wealth of the household and husband’s employment status. In addition to these, lower educational status of the partner affects emotional violence positively.

**Conclusion:**

Substantial proportion of women in Ethiopia continue to suffer from intimate partner violence. Physical and emotional violence were much more common than sexual violence. In the light of determinants, we have reported in here, we recommend empowering women in all realm of life by improving their socio-economic status with focus to their educational and economic status.

## Introduction

Women in the developing world are active participants of both economic and non-economic activities of the household. Their burden is twofold; one they are responsible for raising income for the livelihood of their family and the other, caring for children and the whole family. Their low educational status on top of the existing adverse socio-cultural trends force them to participate in unskilled activities [1]. For example in Ethiopia, especially in the rural setting, women spend more than 14 hours of hard and detrimental physical labor [2]. The devastating thing is that, they are marginalized from the benefits. Compared with men the degree of the problem extends up to less access to land, assets, and other productive resources [3]. On top of these burdens, most of the time women are exposed to violence from their partner [4].

Intimate partner violence (IPV) is a worldwide concern; nearly a third of women experience at least one type of violence from their partners at least once in their life time [4]. The situation is much worse in low income countries [5]. Studies conducted in various African nations uncover that highest proportion of women experience violence from their partners [6,7]. Women experience physical, emotional as well as sexual violence. A number of reasons can be listed to explain why this happens. Even if cultural variables may predict, enormous contribution have been made by socio-economic variables of the society [8].

In legal terms IPV is a breach of human right [4] and exposes women to numerous problems. Evidences indicated that it increases risk of sexually transmitted infections including HIV/AIDS [9,10] and adversely affects the use of maternity health services by the victims [11,12]. Psychological and mental problems were also associated with IPV [13,14]. Furthermore, there were reports of economic, physical and social crisis attributable to IPV [15].

Considering the deep rooted practice and consequences of IPV against women, the sustainable development goals (SDG) has put an agenda of eliminating all forms of violence against women [16]. By the year 2030 every country is expected to be IPV free. Aiming at eliminating IPV lots of initiatives were launched before and after the introduction of SDGs [17]. Governmental and non-governmental sectors’ responsiveness is also expected to be improved as a result of the initiatives.

Protecting women against violence has various implications not only to the women herself but also to the society in general [17]. Improved use of the world’s half population could result in poverty reduction, economic growth, improved societal well-being, and it guarantees sustainable development of a country [18]. The prevailing growth in the globe especially in the developing world doesn’t give special attention to women. Women remain to consistently fall behind men in entrepreneurship, formal labor force participation, credit access, income levels, and ownership and inheritance rights [19]. Under-investing in women limits development, slows down poverty reduction and economic growth. If the situation continues as is the countries cost more to attain development in all dimensions [3].

Studies undertaken in Ethiopia so far, have indicated that more than half of women experience violence from their intimate partners at least once in their life time [7,20,21]. Despite there are studies conducted in the country before, they are limited to some specific geographical location. Moreover, most of the studies haven’t addressed all the dimensions of IPV. Therefore, the aim of this study was to assess intimate partner violence against women in terms of physical, emotional and sexual dimensions and identify associated factors for the three dimensions using a nationally representative data.

## Materials and methods

### Study setting

The largest nation in the Horn of Africa, Ethiopia is a land locked country bounded by Eritrea from the north, Sudan and South Sudan from the West, Kenya from the South, and Somalia and Djibouti from the East. The capital of the country is Addis Ababa, where the head quarter of African Union (AU) is located. Ethiopia has an estimated population of 107.53 million, which ranks 2^nd^ in Africa and 14^th^ in the world. The male to female ratio is almost 50/50 [22–24].

The survey was conducted from January 18, 2016, to June 27, 2016, based on a nationally representative sample that provides estimates at the national and regional levels and for both urban and rural areas.

### Sampling

The data for this study was obtained from the Ethiopian Demographic and Health Survey (EDHS) 2016. The primary objective of survey was to provide up-to-date estimates of key demographic and health indicators.

The sampling frame for the study was a complete list of 84,915 enumeration areas (EAs) created for the 2007 population and housing census. An EA is a geographic area covering on average 181 households. Administratively, Ethiopia is divided into nine regions and two administrative cities. The survey sampling was stratified and selected in two stages. Each region was stratified into urban and rural areas, yielding 21 sampling strata. Samples of EAs were selected independently in each stratum in two stages. Implicit stratification and proportional allocation were achieved at each of the lower administrative levels by sorting the sampling frame within each sampling stratum before sample selection, according to administrative units in different strata, and by applying a probability proportional to size selection at the beginning point of sampling.

In the first stage, a total of 645 EAs (202 in urban areas and 443 in rural areas) were selected with probability proportional to EA size and with independent selection in each sampling stratum. A household listing operation was carried out in all of the selected EAs from September to December 2015. The resulting lists of households served as a sampling frame for the selection of households in the second stage.

In the second stage of selection, a fixed number of 28 households per cluster were selected with an equal probability systematic selection from the newly created household listing. A total of 2750 women aged between 15 and 49 were selected for interview.

### Data collection

All women age 15–49 who were either permanent residents of the selected households or visitors who stayed in the household the night before the survey were eligible to be interviewed. One woman per household was selected for the domestic violence module.

The EDHS data collection tools were divided in to five categories: the Household Questionnaire, the Woman’s Questionnaire, the Man’s Questionnaire, the Biomarker Questionnaire, and the Health Facility Questionnaire. The Woman’s Questionnaire was used to collect information from all eligible women age 15–49. Data on violence against women were collected using this questionnaire. The tool is available online on the DHS program website: https://dhsprogram.com/what-we-do/survey-types/dhs-questionnaires.cfm.

Interviewers selected for the survey used tablet computers to record responses during the interviews. The tablets were equipped with Bluetooth technology to enable remote electronic transfer of files. Transfer of assignment sheets from team editors to interviewers and transfer of completed questionnaires from interviewers to editors were performed electronically.

### Variables

#### Dependent variables

Domestic violence measured in three dimensions: physical violence, emotional violence and sexual violence.

#### Independent variables

Age category; Age difference (husband’s/partner’s age minus the woman’s age); Place of residence; Religion; Wealth; Women’s level of education; Husband/partner's education level; Occupation of the respondent and Husband/partner's occupation were taken as independent variables.

### Data processing and analysis

Relevant variables for this study were extracted form EDHS 2016 data. We used SPSS version 20 (I.B.M Corporation) software for analysis. IPV was measured using three dimensions; namely; physical, emotional and sexual violence. The data were described using frequency and percentage. Bivariate logistic regression was conducted to examine the crude association between each dimension of IPV with each covariate. Finally, multiple logistic regression was conducted to identify independent predictors of each dimension of IPV. Variables with p-value less than 0.05 were considered as significantly associated with violence dimensions. All analyses were adjusted for clusters and sample weights.

## Results

### Description of study participants

A total of 2750 women were selected and 2734 respond for questions related to domestic violence. As compared to other age groups highest proportion of women 686 (25.1%) were between ages 25 and 29. Most of the women, 2347 (85.8%), were residents of rural areas. Close to two third, 1707 (62.4%), of the women were uneducated, only 97 (3.5%) have gone through higher education. The respondents were evenly distributed along the five wealth quintiles with little difference. Relatively poorer quintile takes the highest proportion 584 (21.4%). Only about half of the study participants, 1385 (50.7%), had an occupation. On the other hand, almost all, 2581 (94.4%), of the husbands of the respondents had an occupation (Table 1).

**Table 1:** Socio-demographic and economic characteristics of women, EDHS, 2016.

Variables	Category	Total	Percent
Age category	15–19	137	5.0
	20–24	453	16.6
	25–29	686	25.1
	30–34	577	21.1
	35–39	464	17.0
	40–44	283	10.4
	45–49	134	4.9
Residence	Rural	2347	85.8
	Urban	387	14.2
Level of education	No education	1707	62.4
	Primary	762	27.9
	Secondary	168	6.2
	Higher	97	3.5
Religion	Orthodox	1121	41.0
	Muslin	889	32.5
	Protestant	661	24.2
	Catholic	21	.8
	Traditional	18	.7
	Other	24	.9
Wealth	Poorest	483	17.7
	Poorer	584	21.4
	Middle	574	21.0
	Richer	544	19.9
	Richest	548	20.1
Husband/partner's education level	No education	1225	44.8
	Primary	1075	39.3
	Secondary	229	8.4
	Higher	190	6.9
	Don't know	15	.5
Occupation of the respondent	Not working in the last 12 months	1385	50.7
	Working	1349	49.3
Husband/partner's occupation	Working	2581	94.4
	Not working in the last 12 months	151	5.5
	Don’t know	3	.1

### Experience of intimate partner violence

This analysis revealed that more than one in five (22.5%) of the women included in the study encountered physical and/or emotional violence. However, the prevalence of sexual violence was by far lower, it was 9.6%. Overall, 32.5% of the women in the study reported to have experienced at least one type of IPV. About a quarter of the women (22.5%) have encountered physical or emotional violence. However, only one in ten (9.6%) of the women reported experience of sexual violence.

The women who reported physical violence were also given the opportunity to characterize the physical violence they experienced. We found that 506 (18.5%) of the women have ever been slapped by their husbands/partners. On the other hand, kicking or dragging were the most common sever forms of physical violence committed by intimate partners. The most frequently reported, 493 (18.1%), emotional violence was insulting. 212 (7.8%) of the respondents reported that, they have ever been physically forced into unwanted sex by husband or partner (Table 2).

**Table 2:** Detailed description of intimate partner violence experience among Ethiopian women, EDHS, 2016.

Variable	Frequency	Percent
**Less severe physical violence**		
Ever been slapped by husband/partner	506	18.5
Ever been pushed, shook or had something thrown by husband/partner	321	11.8
Ever been punched with fist or hit by something harmful by husband/partner	184	6.7
Ever had arm twisted or hair pulled by husband/partner	139	5
**Severe physical violence**		
Ever been kicked or dragged by husband/partner	245	8.9
Ever been strangled or burnt by husband/partner	53	2
Ever been threatened with knife/gun or other weapon by husband/partner	44	1.6
**Emotional violence**		
Ever been insulted or made to feel bad by husband/partner	493	18.1
Ever been humiliated by husband/partner	354	13
Ever been threatened with harm by husband/partner	192	7
**Sexual violence**		
Ever been physically forced into unwanted sex by husband/partner	212	7.8
Ever been physically forced to perform sexual acts respondent didn't want to	114	4.2
Ever been forced into other unwanted sexual acts by husband/partner	62	2.2

### Reasons for intimate partner violence

Even more worrying than the fact that a significant proportion of the women experienced physical violence is the attitude the women themselves held about this inappropriate reaction from partners/husbands. It was found that the women tend to justify the violence they experienced with different reasons. More than half (52.1%) of the women agreed that neglecting children could be a justification for violence by their intimate partners. Similarly, about half (49.8%) of the women felt that physical violence by a husband is justified if the wife goes out without telling her husband. Argument with husband, burning food and refusing to have sex were also considered as a justified reason for physical abuse by more than 4 in 10 of the women (Table 3).

**Table 3:** Reasons for intimate partner violence, EDHS, 2016.

Reasons	Frequency	Percent
Beating justified if wife neglects the children	1426	52.1
Beating justified if wife goes out without telling husband	1361	49.8
Beating justified if wife argues with husband	1307	47.8
Beating justified if wife burns the food	1221	44.7
Beating justified if wife refuses to have sex with husband	1104	40.4

### Intimate partner violence by administrative division

**Fig 1:**
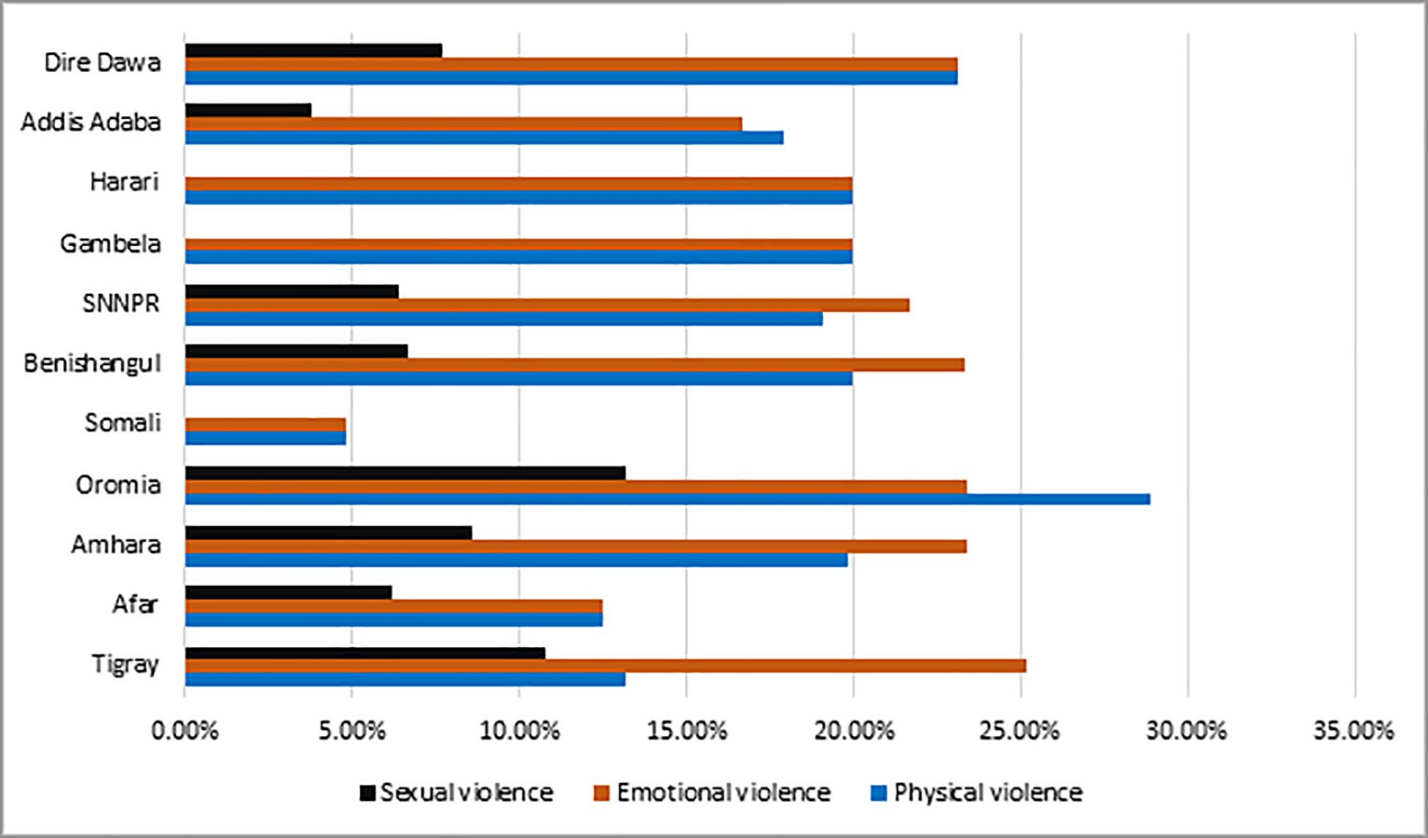
Distribution of intimate partner violence by region and city administration, EDHS, 2016.

We found that, although the degree differs the problem of IPV is an evident concern in all of the nine administrative regional states and two city administrations of Ethiopia. Women in all the regions and city administrations experienced physical, emotional as well as sexual violence with the exception of absence of sexual violence in Somal, Harari and Gambella. In five of the administrative divisions both physical and emotional violence are in equal proportion. On the contrary sexual violence has taken the smallest proportion of other kinds of violence in all the administrative divisions. When we compare regions and city administrations by the three dimensions of violence, Oromia takes the leading position in physical and sexual violence with 28.9% and 13.2% respectively. On the other hand, Tigray has taken the leading position (25.2%) on emotional violence. Somali is a region where all the three dimensions of violence were least frequently encountered (Fig 1).

### Factors associated with intimate partner violence

We fitted three multiple logistic regression models to identify variables significantly associated with intimate partner violence with the three dimensions.

#### Physical violence

The age difference between a woman and her intimate partner favored physical violence [AOR = 0.98, CI 0.96–0.99]. Urban residence was found to have a protective effect against physical violence [AOR = 0.52, CI 0.37–0.75) as compared to rural one. Husband’s education was also found to be another determinant factor. Women who had husbands/partners with no education [AOR = 1.85, CI 1.11–3.09] and primary education [AOR = 1.80, CI 1.08–2.99] where more likely to report physical violence as compared to those whose husband attended higher education (Table 4).

**Table 4:** Factors associated with intimate partner violence, EDHS, 2016.

Variable	Category	Physical violence		Emotional violence		Sexual violence	
		COR [95% CI]	AOR [95% CI]	COR [95% CI]	AOR [95% CI]	COR [95% CI]	AOR [95% CI]
Age difference		0.98 (0.96, 0.99)	0.98 (0.96, 0.99)*	1.02 (1.00, 1.04)	1.02 (1.002, 1.04)*	1.04 (1.01, 1.06)	1.03 (1.01, 1.06)*
Age category	15–19	0.59 (0.31, 1.14)	0.62 (0.32, 1.20)	0.42 (0.22, 0.81)	0.38 (0.19, 0.74)**		
	20–24	1.21 (0.75, 1.95)	1.29 (0.79, 2.10)	0.91 (0.58, 1.44)	0.87 (0.54, 1.39)		
	25–29	1.13 (0.71, 1.79)	1.20 (0.75, 1.92)	0.85 (0.55, 1.32)	0.83 (0.53, 1.31)		
	30–34	1.24 (0.78, 1.98)	1.26 (0.79, 2.02)	1.01 (0.65, 1.57)	0.98 (0.62, 1.54)		
	35–39	1.22 (0.76, 1.97)	1.27 (0.78, 2.05)	1.17 (0.75, 1.83)	1.14 (0.72, 1.81)		
	40–44	1.38 (0.83, 2.27)	1.41 (0.85, 2.35)	0.98 (0.60, 1.59)	0.98 (0.60, 1.61)		
	45–49	1.0	1.0	1.0	1.0		
Residence	Rural	1.0	1.0				
	Urban	0.41 (0.30, 0.57)	0.52 (0.37, 0.75)**				
Religion	Orthodox			1.0	1.0		
	Catholic			0.87 (0.31, 2.45)	1.09 (0.38, 3.16)		
	Protestant			0.83 (0.66, 1.04)	0.79 (0.62, 1.01)		
	Muslin			0.77 (0.62, 0.96)	0.69 (0.55, 0.86)**		
	Traditional			1.33 (0.48, 3.66)	0.99 (0.35, 2.78)		
	Other			3.03 (1.35, 6.79)	3.59 (1.54, 8.39)**		
Wealth	Poorest			1.57 (1.15, 2.14)	1.24 (0.87, 1.76)	4.54 (2.62, 7.87)	4.24 (2.45, 7.37)**
	Poorer			2.00 (1.49, 2.67)	1.65 (1.19, 2.30)**	4.58 (2.68, 7.84)	4.56 (2.66, 7.82)**
	Middle			1.73 (1.28, 2.32)	1.37 (0.99, 1.91)	4.06 (2.36, 6.99)	4.00 (2.32, 6.90)**
	Richer			1.26 (0.92, 1.71)	0.99 (0.70, 1.39)	2.60 (1.47, 4.62)	2.66 (1.5, 4.73)**
	Richest			1.0	1.0	1.0	1.0
Husband occupation	Not working in the last 12 months			1.0	1.0	1.0	1.0
	Working			1.47 (1.03, 2.12)	1.52 (1.04, 2.21)*	1.95 (1.24, 3.06)	1.86 (1.17, 2.94)*
Husband Education	No education	2.60 (1.63, 4.16)	1.85 (1.11, 3.09)*	2.89 (1.77, 4.71)	2.30 (1.33, 3.98)**		
	Primary	2.48 (1.55, 3.98)	1.80 (1.08, 2.99)*	2.76 (1.68, 4.51)	2.61 (1.52, 4.47)**		
	Secondary	1.26 (0.70, 2.27)	1.06 (0.58, 1.93)	1.72 (0.96, 3.10)	1.61 (0.87, 2.98)		
	Higher	1.0	1.0	1.0	1.0		
Constant			0.17		0.11		0.03

*p-value < 0.05

**p-value < 0.01

#### Emotional violence

As the age gap between the woman and her partner increases the probability of emotional violence also increases [AOR = 1.02 CI, 1.002–1.04]. As compared to women in the age group 45–49, those between 15 and 19 were less exposed to emotional violence [AOR = 0.38, CI, 0.19–0.74]. When we look religion wise, Muslims are less likely to be encountered with emotional violence as compared to Orthodox Christian [AOR = 0.69, CI, 0.55–0.86]. Wealth quintile is the other determinant factor for emotional violence. Emotional violence in poorer households is more probable as we compare it to the richest households [AOR = 1.65, CI, 1.19–2.30]. Husbands who have occupation were more likely to commit emotional violence against their wives as compared to those who were not working [AOR = 1.52, CI, 1.04–2.21]. High level of emotional violence was seen in husbands with no education [AOR = 2.30, CI, 1.33–3.98] and primary education [AOR = 2.61, CI, 1.52–4.47] as compared to those who achieved higher education (Table 4).

#### Sexual violence

As the other dimensions described above, an increase in age difference resulted in higher sexual violence [AOR = 1.03, CI, 1.01–1.06]. All of wealth quintile categories increased sexual violence as compared to the richest wealth quintile category. Similarly, as in the case of emotional violence, sexual violence was more likely among women who have husbands with a work as compared to those who have husbands who were not working for the last 12 months [AOR = 1.86, CI, 1.17–2.94] (Table 4).

## Discussion

In response to the highly prevalent and deep rooted level of violence against women in third world countries, various national and international goals including the SDGs were developed [16,17,25]. It became a major focus area of both governmental and non-governmental organizations working on human rights and public health. Even though all these efforts were exerted so far, IPV remains to be a serious concern [6,26]. Mostly, this problems are attributed to legal, health and societal features in addition to personal factors [10,27]. Most of the time violence against women is categorized in to physical, emotional and sexual. Our study has followed this classification to assess intimate partner violence against women in Ethiopia.

In Ethiopia, 23 out of 100 women experienced both emotional and physical violence. Out of these, more than 63% were overlapping, that means there were 63 women who have experienced both physical and emotional violence out of 100 who ever experienced intimate partner violence. On the other hand, one in ten women in Ethiopia experienced sexual violence from her intimate. Nearly 71% and more than 67% of the sexual violence overlapped with physical and emotional violence, respectively.

Almost all earlier studies conducted in Ethiopia from 2009 to 2017 reported higher prevalence of physical, emotional and sexual violence as compared to the findings in our analysis. The proportion of physical violence ranged from eight to more than 60% in studies conducted in western Ethiopia [20], Shimelba refugee camp [28], Kersa district [29] and Awi zone [30]. Similarly, the proportion of emotional violence ranged from 4.8% to 50.3% in various parts of the country [20,29–31]. Sexual violence was also found to be higher in other studies conducted in different parts of the country. The difference from the current study ranges from 26.7% to 52% as compared to studies conducted in East Wollega [20], rural Ethiopia [31] Kersa district of Jimma Zone [29], South Wollo [32], Awi Zone [30] and Wolaita Sodo University [33]. The prevalence of IPV reported from these studies reflect the magnitude of the problem in specific regions of the country. Many of these studies were reported from Oromia, Amhara, and Tigray Regional States where a disaggregated look at the national data (even in our study) shows high prevalence of IPV. However, our finding reflects the cumulative reality of the country including regions where IPV is less frequent, such as Afar and Somali.

From outside of Ethiopia, an earlier study from Iraq reported higher proportions where 52.6% of women experienced emotional, 38.9% physical, and 21.1% sexual violence [34]. On the contrary, intimate partner violence was reported to be lower than our findings in European countries like Azerbaijan, Ukraine and Moldova [35].

Although it is argumentative that women in Ethiopia either accept violence as normal or not [36], they do have various justifications for the violence committed up on them. In the Ethiopian culture most of the time caring for children is considered as the sole responsibility of women (wives) [2,37]. Therefore, neglecting children was the most cited justification for violence against women by their intimate partner. On the other hand, the male partner (husband) is not responsible to let his wife be aware of what he does, or where he goes. However, the woman (wife) especially in rural areas have to inform her husband about where she wants to go [38]. That is why a significant proportion of the victims considered ‘going out without telling her husband’ a justification for violence.

Our analysis indicated that, a one year increase in age gap between the partners (mostly the men older than the women), decreases the likelihood of physical violence by two percent. It has a reverse effect on emotional and sexual violence. An increase in age difference increases the likelihood of emotional and sexual violence 1.02 and 1.03 times respectively. People in close age groups most of the time compete in the same issues. This may lead them to physically express their feelings [39,40]. In support of this finding, a study carried out in Burkina Faso found the physical violence significantly decreases when the age difference is more than 15 years [41]. On the contrary, if the age difference is higher, partners tend to express their feelings with insulting, humiliation or threatening rather than physical harm. On the other hand, sexual desire among two partners increases when their ages are close [42]. With the fact that people do not want to participate in sexual affairs with their elder associates, elder partners may force their partner to have sexual intercourse or any other sexual acts with them.

The major issues that most affect violence are economic status and economic activities. For example, in rich households the level of both emotional and sexual violence were at the minimal level. Therefore, most violence relate to poor economic level and living standards of members of the household. This issues are directly linked to the educational level and place of residence. Majority of well-educated people in Ethiopia have better economic status and live in urban areas [42,43]. On top of what was described above, better educational level and urban residence exposes the people to better information. Our finding revealed that those living in urban areas were less likely to participate in physical violence. Moreover, improved educational status was found to minimize the level of physical and emotional violence against women.

In a study carried out in western Ethiopia educational level of partner/husband was found to be affecting violence positively in line with this study [20]. A review based on studies conducted in Ethiopia has come up with a conclusion implying that occupation, educational status, and residence were determinants of intimate partner violence [21]. On the other hand, a study in Burkina Faso found that residence (rural) negatively affected emotional violence [41].

Occupation of the husband (intimate partner) has also effect on different forms of violence. When husband/intimate partner doesn’t have occupation, they are less likely to be violent. In view of the fact that the husband/intimate partner has no occupation, he becomes dependent on his wife/partner. This dependence forces them to respect their wife’s dignity. This implies that women who socially and economically empowered are less likely to experience any form of domestic violence [44,45].

It should also be understood that, women’s active participation and ownership of economic activities and resources ownership could effect in developed economic status for their family as well as the country in general. A number of studies recommend that respecting the autonomy of women on resources, is a wise way to accelerate development and to eliminate poverty [5,46,47]. In comparison with men, women usually invest a plentiful portion in their families and communities, and distributing resources beyond themselves. That is why countries that achieved gender equality could eliminate poverty. Therefore, empowering women is not a choice of countries, it is a question of existence.

In this study, we have used a large nationally representative sample with which generalization of the findings to the entire country is safe and credible. Moreover, we have addressed all of the critical dimensions of IPV and have identified relevant predictors. However, our study has some limitations. First of all the nature of the study was cross-sectional, by which causal inference is not possible. Second, given the cultural context in Ethiopia, the women interviewed may be afraid and/or ashamed of exposing their personal matters especially when it relates to sexual issues. Therefore, social-desirability bias is very much likely in this study. Third, recall bias should also be taken into account since respondents were asked about their past experiences.

## Conclusion

Despite the fact that success has been attained in reducing the prevalence of IPV in Ethiopia, still substantial proportion of women suffer from at least one form of IPV. Both physical and emotional violence have taken the lead followed by sexual violence. A number of predisposing factors can be listed for this unpleasant reality. Even though some factors have affected some of the dimensions positively, they also had a negative association with other dimensions. For instance age difference has positively affected physical violence while it negatively affected emotional and sexual violence.

Beyond other commitments, focus should be directed to minimizing economic barriers among women. Women should have their own economic base with reasonable degree of autonomy in this regard. Therefore, responsible bodies are supposed to maintain long-lasting investments in the social sectors so that improvements in education, health and social protection are not reversed. Supporting women’s earnings through better access to agricultural inputs and credit lines may be helpful in this regard. It is also essential that all public policies make the effort to ensure social and economic empowerment of women for better autonomy a central theme.

## Ethics consideration

The authors analyzed secondary publicly available data obtained from the DHS program database. There was no additional ethical approval sought by the authors.
